# The Keap1/Nrf2-ARE Pathway as a Pharmacological Target for Chalcones

**DOI:** 10.3390/molecules23071803

**Published:** 2018-07-20

**Authors:** Matheus de Freitas Silva, Letizia Pruccoli, Fabiana Morroni, Giulia Sita, Francesca Seghetti, Claudio Viegas Jr, Andrea Tarozzi

**Affiliations:** 1 PeQuiM-Laboratory of Research in Medicinal Chemistry, Federal University of Alfenas, Jovino Fernandes Sales Avenue, 2600 Alfenas, MG 37130-840, Brazil; matheusfs.1389@gmail.com (M.d.F.S.); cvjviegas@gmail.com (C.V.J.); 2 Department for Life Quality Studies, Alma Mater Studiorum-University of Bologna, Corso d’Augusto 237, 47921 Rimini, Italy; letizia.pruccoli2@unibo.it; 3 Department of Pharmacy and Biotechnology, Alma Mater Studiorum, University of Bologna, Via Irnerio 48, 40126 Bologna, Italy; fabiana.morroni@unibo.it (F.M.); giulia.sita2@unibo.it (G.S.); 4 Department of Pharmacy and Biotechnology, Alma Mater Studiorum, University of Bologna, Via Belmeloro 6, 40126 Bologna, Italy; francesca.seghetti2@unibo.it (F.S.); 5 INBB, National Institute for Biostructures and Biosystems, 00136 Rome, Italy

**Keywords:** chalcones, Nrf2, Keap1, NF-κB, antioxidant activity, anti-inflammatory activity, multi-target activity

## Abstract

Chalcones have shown a broad spectrum of biological activities with clinical potential against various diseases. The biological activities are mainly attributed to the presence in the chalcones of the α,β-unsaturated carbonyl system, perceived as a potential Michael acceptor. Chalcones could activate the Kelch-like ECH-associated protein 1 (Keap1)/Nuclear factor erythroid 2-related factor 2 (Nrf2) pathway through a Michael addition reaction with the cysteines of Keap1, which acts as a redox sensor and negative regulator of Nrf2. This modification allows the dissociation of Nrf2 from the cytoplasmic complex with Keap1 and its nuclear translocation. At this level, Nrf2 binds to the antioxidant response element (ARE) and activates the expression of several detoxification, antioxidant and anti-inflammatory genes as well as genes involved in the clearance of damaged proteins. In this regard, the Keap1/Nrf2–ARE pathway is a new potential pharmacological target for the treatment of many chronic diseases. In this review we summarize the current progress in the study of Keap1/Nrf2–ARE pathway activation by natural and synthetic chalcones and their potential pharmacological applications. Among the pharmacological activities highlighted, anti-inflammatory activity was more evident than others, suggesting a multi-target Michael acceptor mechanism for the chalcones involving key regulators of the Nrf2 and nuclear factor- κB (NF-κB) pathways.

## 1. Introduction

**Figure 1:**
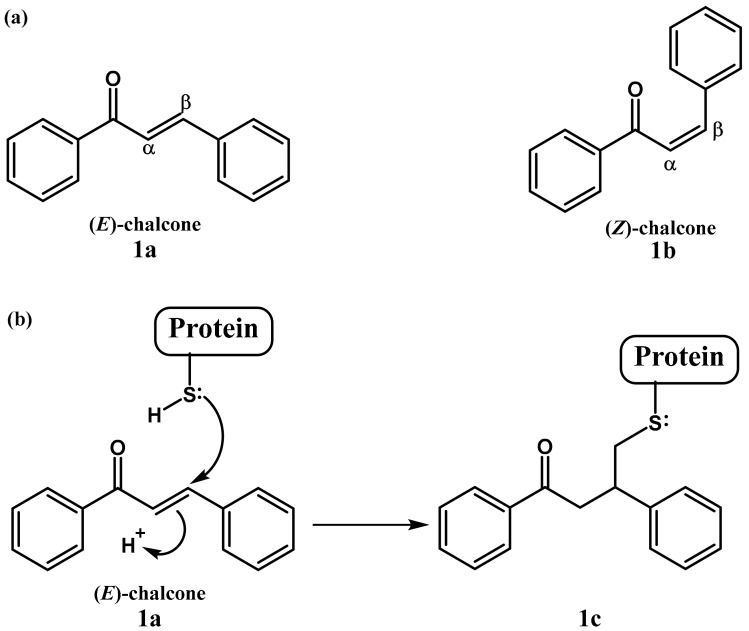
Chalcone structure (**a**) and its Michael addition with cysteine (**b**).

Chalcone or 1,3-diaryl-2-propen-1-one is a common chemical scaffold found in several natural and synthetic chalcone derivates, that exists as either a trans (*E*) or cis (*Z*) isomer (Figure 1a), of which the trans isomer is thermodynamically more stable [1]. Chalcone derivatives have exerted a broad spectrum of biological effects such as antioxidant, antimicrobial, antiprotozoal, antiulcer, antihistaminic, antidiabetic, anti-inflammatory, anticancer and also neuroprotective activities [2,3,4]. These biological activities are mainly attributed to the presence in the chalcones of the α,β-unsaturated carbonyl system, perceived as a potential Michael acceptor [5,6]. This moiety can readily form covalent bonds with nucleophiles such as the sulfhydryl of cysteine residues present in cellular peptides or proteins to obtain the Michael adduct (Figure 1b), which may play an important role in their biological activities [2].

**Figure 2:**
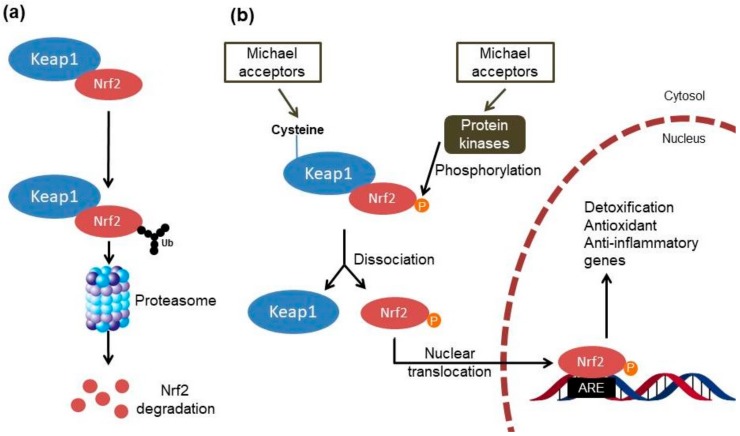
Mechanisms of Nrf2–ARE pathway activation by Michael acceptors. (**a**) Under baseline conditions, Nrf2 forms a complex with Keap1 in the cytosol, which facilitates the ubiquitination and degradation of Nrf2 by the proteasome. (**b**) The dissociation of the Nrf2–Keap1 complex is an essential prerequisite for Nrf2/ARE activation by Michael acceptors that happens through two mechanisms: (i) by modification of the cysteines in Keap1, which leads to conformational changes in this protein and the subsequent release of Nrf2; (ii) by activation of kinases that phosphorylate Nrf2 and thereby free it from Keap1-mediated complex. After nuclear translocation, Nrf2 binds to ARE in the promoter regions of various detoxification, antioxidant and anti-inflammatory genes. Nrf2, Nuclear factor erythroid 2-related factor 2; Keap1, Kelch-like ECH-associated protein 1; ARE, antioxidant response element; Ub, ubiquitin; P, phosphate.

Chalcones and other Michael acceptors could activate the Kelch-like ECH-associated protein 1 (Keap1)/Nuclear factor erythroid 2-related factor 2 (Nrf2) pathway (Figure 2) through covalent bonds with the cysteines of Keap1, which contains at least 27 cysteine residues with different levels of reactivity and acts as a redox sensor and negative regulator of Nrf2 [6,7,8]. This modification reduces the ability of Keap1 to induce ubiquitination and degradation of Nrf2 as well as allowing the dissociation of Nrf2 from the cytoplasmic complex with Keap1 and its translocation into the nucleus [9].

At a nuclear level, Nrf2 binds to the antioxidant response element (ARE) or the electrophile responsive element and activates the expression of several detoxification, antioxidant and anti-inflammatory genes as well as genes involved in the clearance of damaged proteins [9,10]. These genes also encode enzymes that generate the major intracellular antioxidant, glutathione (GSH). Recent studies also reported Keap1-independent mechanisms of Nrf2 regulation (Figure 2), such as the activation of protein kinase signaling cascades by molecules that modify the cellular redox status [11]. In particular, the phosphoinositide 3-kinase/protein kinase B (PI3K/Akt) signaling pathway can activate the Nrf2 signaling through the inhibition of glycogen synthase kinase-3 (GSK-3β) [12]. Furthermore, the p38-mitogen activated protein kinase (p38-MAPK) pathway can phosphorylate Nrf2 and stabilize the association between Nrf2 and Keap1 proteins, promoting its breakdown [11]. Recent studies also showed functional cross-talk between Nrf2 and the nuclear factor-κB (NF-κB); these nuclear factors regulate cellular responses to oxidative stress and inflammation, respectively [13]. Under normal conditions, NF-κB is taken in the cytosol through the interaction with the nuclear κ-B inhibitor (IκBα). The IκB kinase (IKK) activation evoked by tissue damage or infection allows the degradation of IκBα and subsequently the nuclear translocation and activation of NF-κB [14]. Therefore, the Keap1/Nrf2 pathway can exert anti-inflammatory effects through indirect mechanisms by reducing reactive oxygen species (ROS) levels and consequently NF-κB activity, and through direct mechanisms by modulating the transcription of anti- and pro-inflammatory genes, and inhibiting IKK activity through the interaction with Keap1 [13]. In this regard, the chalcones also demonstrate the ability to control inflammation through the inhibition of IKK activity and the DNA binding of NF-κB via a Michael acceptor-related mechanism [15,16,17,18].

The Keap1/Nrf2–ARE pathway involved in oxidative stress and inflammation is a new potential pharmacological target for the treatment of many diseases, including cancer, neurodegenerative diseases, diabetes, airway disorders, cardiovascular disease, inflammatory bowel diseases, rheumatoid arthritis and osteoarthritis [14,19]. In drug discovery, several natural and synthetic compounds have demonstrated the ability to modulate the Keap1/Nrf2–ARE pathway, most of which are activators [20]. Based on the interaction mechanism, Nrf2 activators are classified into electrophilic activators, which act through the modification of Keap1 cysteine residues, and non-electrophilic activators that target the protein–protein interaction interface of Keap1/Nrf2 [20,21]. Recently, some researchers started to identify Nrf2 inhibitors for the treatment of cancers that express a constitutive activation of the Nrf2 function, which contributes to undesired protection of the cancer cells against oxidative stress and xenobiotics. However, further studies are necessary to explain the biological significance of Nrf2 activation in cancer [20].

Among the electrophilic activators of Nrf2, chalcones are mainly soft electrophiles and attract soft nucleophiles like thiols, rather than hard nucleophiles like amino and hydroxyl groups [6]. These highlights suggest that chalcones could be less prone to adverse off-target effects than other electrophilic activators of Nrf2. In this context, compounds with a very high reactivity against Nrf2 may not be the best choice for the design of an ideal Nrf2 activator [22].

Previous reviews have described a broad spectrum of biological activities for chalcones, suggesting a potentially promiscuous target profile [1,2,3,4,5]. Therefore, a knowledge of the different mechanisms of chalcones and direct molecular targets is important for the future clinical development of chalcone compounds. Among the potential molecular targets, the Keap1/Nrf2–ARE pathway is characteristic for its complexity and cross-talks with multifunctional proteins [14], yet has not been systematically reviewed for chalcones. In this review, we will summarize the experimental evidence of direct interactions of natural (Table 1) and synthetic (Table 2) chalcones with the Keap1/Nrf2–ARE pathway and the pharmacological relevance of these interactions. We will not consider the few chalcones that show an ability to inhibit or modulate the Keap1/Nrf2–ARE pathway in cancer prevention and therapy.

## 2. Targeting the Nrf2 Pathway by Natural Chalcones

Liu et al. evaluated the anti-inflammatory properties of trans-chalcone (**1a**), an α,β-unsaturated flavonoid present in several plants and a major precursor of other flavonoids, in lipopolysaccharide (LPS) and interleukin (IL)-6-treated bovine aortic endothelial cells (ECs) [23]. In particular, they recorded the ability of **1a** to reduce the adhesion of monocyte THP-1 cells, to stimulate the overexpression of the adhesion molecule ICAM-1, an early event of the inflammatory process, and to inhibit the activation of STAT3 and NF-κB in IL-6- and LPS-treated ECs. Moreover, **1a** showed a higher activity than other flavonoids which lack the α,β-unsaturated carbonyl group, suggesting that this functional group was crucial for the anti-inflammatory activity recorded. In this regard, the authors also highlighted that the anti-inflammatory response elicited by **1a** could be ascribed to its ability to transiently deplete intracellular GSH levels, emphasizing that the thiol-dependent redox state may influence the activity of this chalcone. Further, they determined that **1a** upregulated nuclear Nrf2 levels, ARE-luciferase, thioredoxin reductase 1 (TrxR1) and heme oxygenase-1 (HO-1) activities, probably in response to the electrophilic stress generated. Taken together, these results support the contribution of the α,β-unsaturated carbonyl moiety of **1a** to its anti-inflammatory properties at endothelial level. Martinez et al. also evaluated the antioxidant and anti-inflammatory activity of **1a** in a model of ultraviolet B radiation skin damage in hairless mice [24]. They reported that topical administration of **1a** reduced several parameters of skin inflammation and oxidative stress, such as skin edema, myeloperoxidase (MPO), lactoperoxidase (LPO) and matrix metallopeptidase 9 (MMP-9) activities, as well as tumor necrosis factor-α (TNF-α) production and cyclooxygenase-2 (COX-2) expression. Nrf2, HO-1, GSH peroxidase 1 (GPX1) and GSH reductase (GR) expressions, chloramphenicol acetyltransferase (CAT) activity and GSH levels were also enhanced at skin level by treatment with **1a**. On the basis of these findings, the authors suggest further studies to confirm a therapeutic approach with **1a** for the treatment of UVB radiation skin damage.

Lee et al. investigated the anti-inflammatory activity of xanthohumol (**2**), a polyphenol chalcone from hops (*Humulus lupulus* L.), in microglial BV2 cells [25]. Chalcone **2** reduced the release of pro-inflammatory mediators including nitric oxide (NO), IL-1β, and TNF-α, as well as the activation of the NF-κB pathway in LPS-stimulated BV2 cells. Moreover, **2** triggered the activation of the Nrf2–ARE pathway, the transcription of NAD(P)H quinone dehydrogenase 1 (NQO1) and HO-1 and the increase of GSH levels. Yao et al. also demonstrated that **2** activated the nuclear translocation of Nrf2 to confer neuroprotection against oxidative damage in neuronal PC12 cells [26]. Treatment of PC12 cells with **2** upregulated several cytoprotective genes, as well as the corresponding proteins, including GSH, HO-1, NQO1, thioredoxin 1 (Trx1), and TrxR1. This strengthening of the intracellular antioxidant defense system was associated with prevention of ROS accumulation and neuronal death induced by hydrogen peroxide (H_2_O_2_) or 6-hydroxydopamine (6-OHDA) in PC12 cells. Interestingly, the authors recorded that both the hydrogenation of **2** and Nrf2-knockdown in PC12 cells abolished its neuroprotective effect, indicating that the α,β-unsaturated ketone structure in **2** and the activation of Nrf2 are key determinants for the neuroprotection of **2**. More recently, Brodziak-Jarosz et al. confirmed the ability of **2** to form a covalent bond with the sensor protein Keap1, an event that triggers Nrf2 activation, using a click chemistry approach and AREc32 cells [27]. They showed a weaker electrophilicity for **2**, suggesting its more selective interaction with the most nucleophilic thiols. Further, they did not disregard the fact that some noncovalent protein interactions can contribute to target selectivity of **2**. Taken together, these findings at microglial and neuronal level suggested the potential use of **2** to prevent inflammatory and oxidative damage in the brain.

Kim et al. evaluated the ability of Licochalcone E (**3**), a retrochalcone derivative identified in *Glycyrrhiza-inflata*, to activate the Nrf2/ARE pathway in several in vitro and in vivo models of gliosis and neurodegeneration [28]. Chalchone **3** decreased the LPS-induced inflammatory responses in BV2 cells, and also protected neuronal SH-SY5Y cells from 6-OHDA cytotoxicity. In similar experimental conditions, **3** was shown to activate the Nrf2–ARE system and up-regulate NQO1 and HO-1 downstream. Both the cytoprotective activity and the up-regulation of HO-1 and NQO1 by **3** were also established in an in vivo 1-methyl-4-phenyl-1,2,3,6-tetrahydropyridine (MPTP) animal model. The contribution of Nrf2 to the anti-inflammatory and cytoprotective activities of **3** was confirmed using siRNA-mediated Nrf2-silencing cells as well as in the presence of a specific inhibitor of HO-1 or NQO1.

Liu et al. evaluated the cytoprotective effects of Hydroxysafflor yellow A (**4**), a chalcone glycoside isolated from *Carthamus tinctorius*, against ischemia/reperfusion (I/R) injury in cardiomyocyte H9c2 cells [29]. In this regard, chalcone glycoside **4** raised the HO-1 activity, Akt phosphorylation and Nrf2 nuclear translocation, and, most importantly, reduced the H9c2 cardiomyocyte death evoked by I/R. In these experimental conditions, the inhibition of the PI3K/Akt pathway partially abolished the results obtained on HO-1, Nrf2 and cardiomyocyte death, suggesting that the cardioprotective effect recorded with **4** depends on different mechanisms, including the activation of the PI3K/Akt/Nrf2 signaling pathway. Subsequently, Hu et al. and Chen et al. corroborated the ability of **4** to protect H9c2 cells under different ischemic-like conditions, through Nrf2 pathway activation [30,31]. They confirmed these findings on ischemia-reperfusion and isoproterenol-induced myocardial injury in rats, respectively. Interestingly, in the same in vivo models, **4** also showed synergistic cardioprotective effects with Danshensu and acetyl-11-keto-β-boswellic acid on the Nrf2 pathway. Taken together, these highlights suggest that **4** may improve the prognosis of myocardial infarction after post-ischemia reperfusion.

Lee et al. showed the ability of 4,2′,5′-trihydroxy-4′-methoxychalcone (**5**), a chalcone isolated from the heartwood of *D. odorifera*, to inhibit the Nrf2/HO-1 pathway as well as several parameters of inflammation, including NO production, COX-2 and nitric oxide synthase (iNOS) expression, and TNF-α and IL-1β release in LPS-stimulated murine peritoneal macrophages [32]. These results indicate that **5** may be considered for further development for the treatment of a variety of inflammatory diseases.

Ajiboye et al. investigated the capability of lophirones B (**6**) and C (**7**), chalcone dimers present in *Lophira alata*, to induce expressions and activities of cytoprotective enzymes at liver level [33]. Chalcone **6** and **7** administrations to rats increased and decreased the levels of nuclear Nrf2 and cytoplasmic Keap1, respectively, leading to the enhancement of several cytoprotective [GSH S-transferase (GST), NQO1, erythropoietin-producing human hepatocellular receptor (EPH) and UDP glucuronosyltransferase family 1 member A1 (UGT1A1)] and antioxidant [superoxide dismutase (SOD), CAT, GPX and GR] enzymes. These data indicate the detoxification potentials of **6** and **7** at liver level.

Wang et al. evaluated the anti-inflammatory activity of various flavonoids derived from the roots of *Glycyrrhizae uralensis* in macrophage RAW264.7 cells stimulated by LPS [34]. Among these flavonoids, isoliquiritin (**8**) and isoliquiritigenin (**9**) showed a higher inhibition of the inflammatory responses mediated by RAW264.7 cells than liquiritigenin. A mechanistic approach also showed the ability of **8** and **9** to activate Nrf2 and induce UGT1A1 and NQO1 enzymes in RAW264.7 cells. The authors further found that both **8** and **9** induced HO-1 expression regardless of Nrf2 expression in hepatic HepG2-C8 cells. With regard to NF-κB signaling, they recorded an inhibition of IκBα degradation and phosphorylation only for **9**. Zeng et al. investigated the ability of **9** to control NF-κB and NLRP3 inflammasome pathways during intracerebral hemorrhage in rats [35]. Chalcone **9** suppressed NF-κB and NLRP3 inflammasome components and activated Nrf2-mediated antioxidant system. Moreover, **9** counteracted several parameters of neurological damage. Taken together, these findings suggest that **9** can modify several components of the inflammatory processes at different tissue and organ levels including brain.

Han et al. showed that isosalipurposide (**10**), a chalcone isolated from *Corylopsis coreana* Uyeki, activates the Nrf2–ARE signaling pathway via the phosphorylation of extracellular signal-regulated kinase 1 ERK1/2 and AMP-activated protein kinase (AMPK) in human hepatic HepG2 cells [36]. They also demonstrated that Nrf2 activation elicited by **10** indicated its ability to increase the protein levels of glutamate cysteine ligase (GCL) and HO-1, as well as intracellular GSH levels, resulting in increased resistance of HepG2 cells against tert-butylhydroperoxide-induced oxidative damage.

Ma et al. reported that among the components of *Carthamus tinctorius*, Safflower yellow B (**11**), a quinochalcone glycoside, protects HepG2 cells against the oxidative damage induced by H_2_O_2_ [37]. In particular, **11** reduced H_2_O_2_-dependent intracellular ROS production, malondialdehyde (MDA) formation and cell damage, and increased antioxidant enzyme activity, such as GPX and SOD. The authors also demonstrated that **11** induced the activation of Nrf2/HO-1 and GPX and SOD levels by triggering the Akt pathway.

Pinner et al. compared the ability of flavokawains A (**12**) and B (**13**), methoxylated chalcones from Kava (*Piper methysticum*), to induce an adaptive cellular response in HepG2 cells [38]. Both methoxylated chalcones activated the transcription factors Nrf2, increasing the expression of antioxidant and heat shock (Hsp) response genes, in HepG2. In particular, **12** and **13** increase HO-1, glutamate-cysteine ligase complex (GCLC), heat shock protein family A (Hsp70) member 1A (HSPA1A) and Dnaj heat shock protein family (Hsp40) member A4 (DNAJA4) gene expression as well as intracellular total GSH levels. In addition, pre-treatment of HepG2 cells with **12** and **13** prevented cell death induced by subsequent treatment with H_2_O_2_; interestingly, **12** was more effective than **13**. On the basis of the antioxidant, cytoprotective and toxic effects recorded with **12**, the authors suggest its potential use as a chemopreventive agent.

Martinez et al. investigated the potential dermatological use of hesperidin methyl chalcone (**14**), an antioxidant flavonoid, to prevent and/or reduce UVB irradiation-induced skin inflammation and oxidative stress in hairless mice [39]. The topical administration of **14** protected the skin from UVB damage, enhancing endogenous antioxidant systems, including CAT activity and GSH levels, as well as GPX1, GR, Nrf2 and HO-1 mRNA expression, and inhibited the production of pro-inflammatory molecules, such as TNF-α and IL-1β. These data suggest that **14** is a promising chalcone derivative for protecting the skin from the damage of UVB irradiation.

Peng et al. reported the capability of cardamonin (**15**), a chalcone isolated from *Alpinia katsumadai*, to reduce oxidative stress and cell death induced by H_2_O_2_ and 6-OHDA in neuronal PC12 cells [40]. Treatment of PC12 cells with **15** increased Nrf2 nuclear translocation, resulting in the enhancement of total GSH levels and HO-1, NQO1, Trx1, TrxR1, GCLC and glutamate-cysteine ligase modifier (GCLM) gene expression. The silencing of Nrf2 expression in PC12 cells abolished the neuroprotective effects of **15**, indicating that its neuroprotection may be mediated by Nrf2 activation. Other authors evaluated the particular ability of **15** to also upregulate Nrf2-regulated selenoenzymes, such as GPX2 and TrxR1 in intestinal Caco-2 cells [41].

Wang et al. identified butein (**16**), a flavonoid chalcone found in *Toxicodendron vernicifluum*, as a novel inducer of HO-1 expression in adipocytes in vitro and in vivo [42]. Chalcone **16** increased HO-1 expression in adipocyte 3T3-L1 cells and promoted Keap1 degradation as well as Nrf2 nuclear translocation. These findings were reversed by a p38-MAPK inhibitor, suggesting its involvement in **16** activation of Nrf2 in adipocytes. In addition, the enhancement of HO-1 decreased ROS formation and the process of adipogenesis. The authors also recorded the ability of **16** to inhibit adipose hypertrophy and adipose tissue inflammation in C57BL/6 mice fed with a high-fat diet.

## 3. Targeting the Nrf2 Pathway by Synthetic Chalcones

Alcaraz et al. evaluated the role of HO-1 and Nrf2 in the anti-inflammatory activity of 3′, 4′, 5′, 3, 4, 5-hexamethoxy-chalcone (**17**), a synthetic chalcone already characterized, at least in part, for its anti-inflammatory properties against NO production and iNOS induction [43]. In RAW264.7 cells stimulated with LPS, chalcone **17** inhibited NF-κB translocation into the nucleus, DNA binding and transcriptional activity, along with activation of Nrf2 and HO-1. In particular, the authors demonstrated that in the same experimental conditions **17** generated an increase of ROS without toxicity, responsible for Nrf2 and HO-1 activation, suggesting a different mechanism of action from antioxidant chalcones.

Lee et al. previously showed a potent anti-inflammatory effect of 2′,4′,6′-tris(methoxymethoxy) chalcone (**18**), a synthetic chalcone derivative, in LPS-stimulated RAW264.7 cells. Subsequently, they also evaluated the anti-inflammatory effects of **18** in a trinitrobenzene sulfonic acid-induced colitis model and the involvement of the Nrf2/HO-1 pathway in the anti-inflammatory responses in intestinal HT-29 cells [44]. Treatment of mice with **18** reduced several markers of mucosal inflammation, such as IL-1β and TNF-α and mucosal ulceration induced by trinitrobenzene sulfonic acid. Chalcone **18** also prevented TNF-α-induced inflammation in HT-29 cells through the nuclear translocation of Nrf2 and a consequent increase of HO-1 expression. In particular, **18** inhibited TNF-α-induced NF-κB p65 translocation directly and indirectly by HO-1, without affecting IκBα degradation. Further, the authors demonstrated that upstream ERK1/2 and p38 phosphorylation induced by **18** was required to activate the Nrf2/HO-1 pathway. These findings suggest a potential use of **18** in the treatment of intestinal inflammatory diseases.

During development of optimal anti-inflammatory chalcones, Park et al. synthesized another two new chalcone derivatives (**19** and **20**) that showed an ability to inhibit LPS-stimulated NO and TNF-α production in RAW264.7 cells, a typical early inflammatory response [45,46]. In this regard, mechanistic studies demonstrated that both chalcones decreased NO production in RAW264.7 cells via simultaneous induction of the Nrf2/HO-1 pathway and inhibition of the activator protein 1 (AP-1) activation.

Jin et al. synthesized an additional synthetic **21** derivative and investigated its anti-inflammatory effects in RAW264.7 cells [47]. Chalcone **21** inhibited NO release and iNOS expression in LPS-stimulated RAW264.7 cells via down-regulation of the inflammatory p38/c-Jun N-terminal kinase (JNK) pathway as well as inhibition DNA binding of NF-κB. They also demonstrated the induction of the protective Nrf2/HO-1 pathway. In particular, the activation of this pathway required a transient depletion of GSH, suggesting that early impairment of the redox state is necessary to trigger Nrf2 activation. Based on these findings, the authors suggested the evaluation of the anti-inflammatory properties of **21** in an in vivo model. Taken together, the results support the suggestion that chalcone derivatives **19**, **20** and **21** could be potential agents for the treatment of inflammation-associated diseases.

Shibuya et al. investigated the mechanisms that underlie the anti-ulcer activity of 20-carboxymethoxy-4,4-bis(3-methyl-2-butenyloxy)chalcone (**22**) in gastric epithelial RGM-1 cells [48]. In particular, the authors demonstrated that vascular endothelial growth factor (VEGF) induction by **22** treatment is associated with Nrf2/HO-1 pathway activation in gastric cells. These results show that the angiogenetic action of **22**, through VEGF production, can contribute to its ability to promote ulcer healing.

Kachadourian et al. assessed the mechanisms by which 2′,5′-dihydroxychalcone (**23**) increases cellular GSH levels using MCF-7/AREc32 cells, a cell line stably expressing a luciferase reporter gene driven by ARE [49]. In particular, they showed that an increase of Nrf2–ARE activity and GSH levels induced by **23** could be ascribed to a combination of ROS-dependent and -independent pathways using several inhibitors of ROS and MAPK pathways. These authors suggest other studies to evaluate the contribution of **23** on possible synergistic effects on the activation of the Nrf2–ARE transcriptional pathway.

Kumar et al. evaluated the potency of a chalcone derivative series to activate the expression of Nrf2 as well as antioxidant genes including GCLM, NQO1 and HO-1 [50]. The structure–activity relationship analysis showed that chalcone derivatives with a trifluoromethyl substitution at ortho position on ring B were the most active compounds in human bronchial epithelial Beas-2B cells. Among these chalcones, 2-trifluoromethyl-2′-methoxychalone (**24**) showed the highest ability to increase all the antioxidant genes. Interestingly, a similar profile of gene induction was also recorded in the small intestine of mice treated by gavage with **24**. Further, the authors reported that the activation of Nrf2 by **24** was independent of redox status changes, but they did not determine whether this phenomenon could be ascribed to its ability to directly modify the thiols of Keap1. In other studies [51], chalcone **24** reduced inflammation and airway hyper responsiveness in a mouse model of allergic asthma and prevented dermal fibrosis in fibroblasts of subjects with systemic sclerosis, and in a mouse model of fibrosis through the activation of Nrf2 [51,52].

Wu et al. synthesized the chalcone **25** based on chalcone derivatives (*E*)-3-(4-hydroxy-3-methoxyphenyl)-1-(4-methoxyphenyl) prop-2-en-1-one with known anti-inflammatory properties [53]. Chalcone **25** increased the expression of Nrf2-dependent antioxidant genes, such as GCLC and HO-1, and their corresponding proteins, together with counteracting neuronal death induced by H_2_O_2_ in PC-12 cells. These preliminary data suggest that **25** could prevent oxidative stress-related neurodegenerative disease by activating the Nrf2–ARE pathway.

From a series of α-X-substituted 2′,3,4,4′-tetramethoxychalcones, Rucker et al. recorded the highest ability to activate the Nrf2/HO-1 pathway and to inhibit NF-κB, with corresponding effects on their respective transcriptional gene products for α-X-substituted **26**, **27**, **28** (X = CF_3_, Br and Cl) in several in vitro models, including MCF-7/AREc32 cells, RAW264.7 cells, human macrophages and cervical HeLa cells [54]. The results emphasize that chemical-fine tuning of the Michael acceptor site is necessary to improve the anti-inflammatory activity of chalcones. More recently, Jobst et al. demonstrated the ability of **26** and **27** to inhibit the Janus kinase/signal transducers and activators of transcription (JAK/STAT) signaling in IL-3 dependent Ba/F3 and HeLa cells, suggesting that both chalcones can also interact with different kinases, expanding their pharmacological effect profile [55].

Lounsbury et al. synthesized other pyridyl chalcones, introducing pyridine nitrogen atoms into various positions of the two aromatic rings of **29** [56]. Pyridyl chalcone **29** enhanced HO-1 expression 3-fold higher than the non-heterocyclic chalcone **29** in bronchial epithelial Beas-2B cells. This antioxidant activity in bronchial cells was confirmed in vivo on lungs of mice treated orally with **29**.

Kaufmann et al. demonstrated that among a series of α-X-substituted 2′,3,4,4′-tetramethoxychalcones (α-X-TMCs, X = H, F, Cl, Br, I, CN, Me, p-NO_2_-C_6_H_4_, Ph, p-OMe-C_6_H_4_, NO_2_, CF_3_, COOEt, COOH), able to activate the Nrf2/HO-1 pathway and inhibit NF-κB downstream target genes, only E-α-(4-methoxyphenyl)-2′,3,4,4′-tetramethoxychalcone (E-α-p-OMe-C_6_H_4_-TMC) (**30**) showed a significant cytoprotective effect against staurosporine-induced toxicity in RAW264.7 cells [57]. In this regard, the cytoprotective effect of **30** may be partly related to both the non-toxic activation and inhibition of the Nrf2/HO-1 and NF-κB pathways, respectively. The authors also evaluated the anti-inflammatory activity of **30** in RAW264.7 cells treated with LPS [58]. The results confirmed the ability of **30** to control several inflammatory parameters including the release of proinflammatory cytokines IL-1β, IL-6 and monocyte chemoattractant protein 1 (MCP-1), remarkably through the activation of Nrf2/HO-1.

Zhong et al. previously found that a novel chalcone **31** was able to inhibit the inflammatory response in macrophages treated with LPS [59]. Subsequently, they also demonstrated the ability of **31** to decrease the cardiac hypertrophy, fibrosis and apoptosis induced by high glucose and streptozocin (STZ) in H9c2 cells and mice, respectively. In both models, the anti-inflammatory and cytoprotective effects of **31** were associated with NF-κB nucleus entry blockage and Nrf2 activation. The authors suggest that **31** can be a potential cardioprotective compound, targeting NF-κB and Nrf2 in diabetic cardiomyopathy (DMC).

Rampa et al. demonstrated the ability of the leaving fragment (**32**) during ChEs’ carbamoylation by(E)-3-(((3-(3-hydroxy-4-(3-(3,4,5-trimethoxyphenyl)acryloyl)phenoxy)propyl)(methyl)amino)methyl)phenyl methylcarbamate to exert indirect antioxidant activity in terms of intracellular GSH increase as well as the ability to counteract the neurotoxicity elicited by amyloid oligomers in neuronal SH-SY5Y cells [60]. On the basis of the electrophilic *trans*-α,β-unsaturated carbonyl framework of **32**, the authors hypothesize that this chalcone derivative could increase the antioxidant endogenous defense via the Keap1/Nrf2¬–ARE pathway.

Lee et al. reported a novel morpholine-containing chalcone (**33**) that induces Nrf2 nuclear translocation and NQO1, HO-1, GCL expression, and protease activities of proteasome subunits PSMB5, PSMB7, PSMB8 and PSMA1, as well as lowering α-synuclein aggregation in dopaminergic CATH.a cells [61]. In MPTP mice, chalcone **33** activated a similar Nrf2 pathway and prevented both impaired motor activity and nigrostriatal neurodegeneration. Therefore, they suggest a potential employment of **33** toward development of the therapy for Parkinson’s disease.

From a series of Aza resveratrol–chalcone derivatives, You et al. selected the chalcone **34** that previously showed the highest anti-inflammatory activity against the inflammation induced by LPS in macrophages, to evaluate its ability to control the oxidative stress and inflammation in in vitro and in vivo models of DCM [62,63]. Chalcone **34** reduced the fibrosis, hypertrophy and apoptosis induced by high glucose and STZ in H9c2 cells and mice, respectively. In both DCM models, the treatment with **34** was remarkably associated with NF-κB nucleus entry inhibition and Nrf2 activation, promoting anti-inflammatory and antioxidant effects. The results indicated that **34** can be a promising cardioprotective agent in the treatment of DCM by inhibiting inflammation and alleviating oxidative stress. This study showed that NF-κB and Nrf2 could be therapeutic targets of chalcones to control the pathogenesis of DCM.

## 4. Conclusions

**Figure 3:**
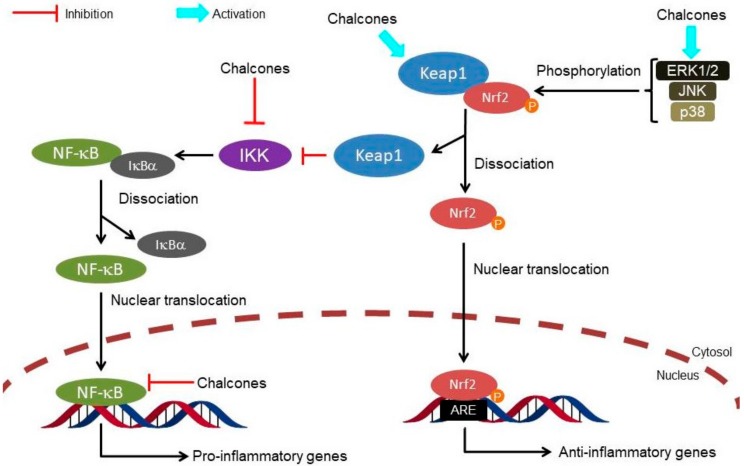
Multi-target interaction of chalcones on cross-talk between Nrf2 and NF-κB response pathways involved in inflammation. Nrf2, Nuclear factor erythroid 2-related factor 2; Keap1, Kelch-like ECH-associated protein 1; ARE, antioxidant response element; P, phosphate; ERK1/2, extracellular signal-regulated kinase; JNK, c-Jun N-terminal kinase; p38-MAPK, p38-mitogen activated protein kinase; NF-κB, nuclear factor-κB; IκBα, nuclear κ-B inhibitor; IKK, IκB kinase.

The present review indicates that chalcones mainly exert antioxidant, anti-inflammatory, antidiabetic, cardioprotective, neuroprotective and cytoprotective activities through Keap1/Nrf2–ARE pathway activation. Among these activities, anti-inflammatory activity was more evident than other pharmacological activities, suggesting a multi-target Michael acceptor mechanism for the chalcones, involving key regulators of the Nrf2 and NF-κB pathways, such as Keap1, IKK, NF-κB, ERK1/2, JNK and p38-MAPK (Figure 3). The multi-target effects of the chalcones on cross-talk between the Nrf2 and NF-κB response pathways can synergize in the overall anti-inflammatory effect. Considering that most chronic diseases are multifactorial and involve different etiological target, this multi-target approach may be useful for therapy [20]. However, the critical feature of chalcones is their potential promiscuity, or poor selectivity, leading to off-target interactions and hence undesirable side effects and compromised potency [4,64,65]. In this regard, many studies selected in this review recorded data of chalcones from phenotypic cell-based screenings combined with animal-based models, without employing biochemical studies to evaluate the selectivity of the chalcones against the cysteine residues on the protein target. In conclusion, a better understanding of the relationship between Nrf2 activation and the overall therapeutic effects as well as interaction manners and therapeutic relevance of such interaction should facilitate further development of chalcone-based therapeutic agents.

## Figures and Tables

**Table 1:** Natural chalcones as Nrf2 inducers.

Compound	Active Concentration Against Nrf2	Target Disease	Study Model	Activity	Ref.
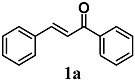 (*E*)-chalcone (*trans*-chalcone)	10–50 μM	Atherogenesis	Aortic endothelial cells	Antioxidant and anti-inflammatory	[23]
	1% *w*/*w* ^a^	UVB skin damage	Hairless mice	Antioxidant and anti-inflammatory	[24]
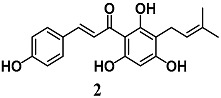 (E)-3-(4-hydroxyphenyl)-1-(2,4,6-trihydroxy-3-(3-methylbut-2-en-1-yl)phenyl)prop-2-en-1-one (Xanthohumol)	5 μg/mL	Neuroinflammation	Microglial BV2 cells	Anti-inflammatory	[25]
	0.5 μM	Parkinson’s disease	Neuronal PC12 cells	Antioxidant and neuroprotective	[26]
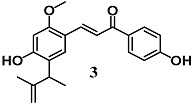 (*E*)-3-(4-hydroxy-2-methoxy-5-(3-methylbut-3-en-2-yl)phenyl)-1-(4-hydroxyphenyl)prop-2-en-1-one (Licochalcone E)	5 μM	Parkinson’s disease	Microglial BV2 and neuronal SH-SY5Y cells	Antioxidant and neuroprotective	[27,28]
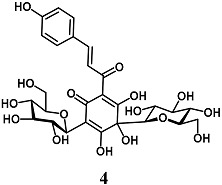 3,4,5-trihydroxy-2-((*E*)-3-(4-hydroxyphenyl)acryloyl)-4-((2*R*,3*R*,4*S*,5*S*,6*R*)-3,4,5-trihydroxy-6-(hydroxymethyl)tetrahydro-2*H*-pyran-2-yl)-6-((2S,3R,5S,6R)-3,4,5-trihydroxy-6-(hydroxymethyl)-tetrahydro-2*H*-pyran-2-yl)cyclohexa-2,5-dien-1-one (Safflor yellow A)	20 μM	Myocardial infarction	Cardiomyocyte H9c2 cells	Cytoprotective	[29]
	10 μM 100 mg/kg ^b^	Myocardial infarction	Cardiomyocyte H9C2 cells Rats	Antioxidant and cytoprotective	[30]
	80 μM	Myocardial infarction	Cardiomyocyte H9c2 cells	Cytoprotective	[31]
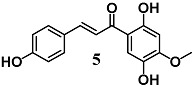 (*E*)-1-(2,5-dihydroxy-4-methoxyphenyl)-3-(4-hydroxyphenyl)prop-2-en-1-one	40 μM	Inflammatory disease	Peritoneal macrophages	Anti-inflammatory	[32]
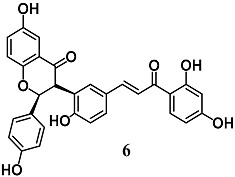 (2*S*,3*R*)-3-(5-((*E*)-3-(2,4-dihydroxyphenyl)-3-oxoprop-1-en-1-yl)-2-hydroxyphenyl)-6-hydroxy-2-(4-hydroxyphenyl)chroman-4-one (Lophirones B) 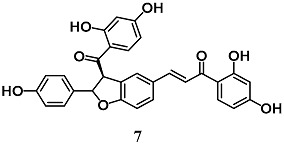 (*E*)-3-((3*S*)-3-(2,4-dihydroxybenzoyl)-2-(4-hydroxyphenyl)-2,3-dihydrobenzofuran-5-yl)-1-(2,4-dihydroxyphenyl)prop-2-en-1-one (Lophirones C)	20 mg/Kg ^b^	Liver diseases	Rats	Antioxidant and detoxification	[33]
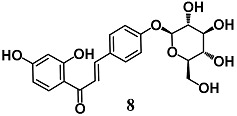 (*E*)-1-(2,4-dihydroxyphenyl)-3-(4-(((2*S*,3*R*,4*S*,5*S*,6*R*)-3,4,5-trihydroxy-6-(hydroxymethyl)tetrahydro-2*H*-pyran-2-yl)oxy)phenyl)prop-2-en-1-one (Isoliquiritin) 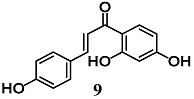 (*E*)-1-(2,4-dihydroxyphenyl)-3-(4-hydroxyphenyl)prop-2-en-1-one (Isoliquiritigenin)	25–50 μM	Inflammatory disease	Macrophage RAW 264.7 and Hepatic HepG2-C8 cells	Anti-inflammatory	[34]
	20 mg/Kg ^c^	Intracerebral hemorrhage	Rats	Antioxidant and anti-inflammatory	[35]
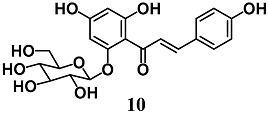 (*E*)-1-(2,4-dihydroxy-6-(((2*S*,3*R*,5*S*,6*R*)-3,4,5-trihydroxy-6-(hydroxymethyl)tetrahydro-2H-pyran-2-yl)oxy)phenyl)-3-(4-hydroxyphenyl)prop-2-en-1-one (isosalipurposide)	10–100 μM	Oxidative stress	Hepatic HepG2 cells	Antioxidant and cytoprotective	[36]
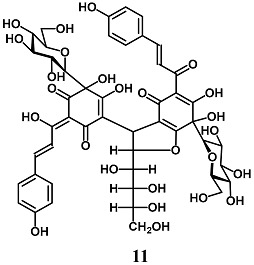 (*Z*)-4-(6,7-dihydroxy-5-((*E*)-3-(4-hydroxyphenyl)acryloyl)-4-oxo-2-((1*S*,2*R*,3*R*)-1,2,3,4-tetrahydroxybutyl)-7-((2*R*,3*R*,4*S*,5*S*,6*R*)-3,4,5-trihydroxy-6-(hydroxymethyl)tetrahydro-2*H*-pyran-2-yl)-2,3,4,7-tetrahydrobenzofuran-3-yl)-5,6-dihydroxy-2-((*E*)-1-hydroxy-3-(4-hydroxyphenyl)allylidene)-6-((2*R*,3*R*,4*S*,5*S*,6*R*)-3,4,5-trihydroxy-6-(hydroxymethyl)tetrahydro-2*H*-pyran-2-yl)cyclohex-4-ene-1,3-dione (Safflor yellow B)	100–150 nM	Oxidative stress	Hepatic HepG2 cells	Antioxidant	[37]
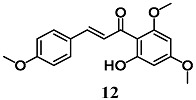 (*E*)-1-(2-hydroxy-4,6-dimethoxyphenyl)-3-(4-methoxyphenyl)prop-2-en-1-one (Flavokawains A) 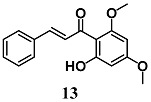 (*E*)-1-(2-hydroxy-4,6-dimethoxyphenyl)-3-phenylprop-2-en-1-one (Flavokawains B)	20–100 μM	Oxidative stress	Hepatic HepG2 cells	Antioxidant and cytoprotective	[38]
	10–20 μM				
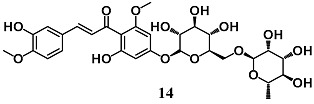 (*E*)-3-(3-hydroxy-4-methoxyphenyl)-1-(2-hydroxy-6-methoxy-4-(((2*R*,3*R*,4*S*,5*S*,6*R*)-3,4,5-trihydroxy-6-((((2*R*,3*R*,4*R*,5*R*,6*S*)-3,4,5-trihydroxy-6-methyltetrahydro-2*H*-pyran-2-yl)oxy)methyl)tetrahydro-2*H*-pyran-2-yl)oxy)phenyl)prop-2-en-1-one Hesperidin methyl chalcone (HMC)	1% *w*/*w* ^a^	UVB skin damage	Hairless mice	Antioxidant and cytoprotective	[39]
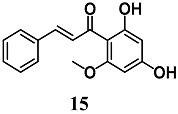 (*E*)-1-(2,4-dihydroxy-6-methoxyphenyl)-3-phenylprop-2-en-1-one (Cardamonin)	50 μM ^d^	Oxidative stress	Intestinal Caco-2 cells	Antioxidant	[40]
	10 μM	Parkinson’s disease	Neuronal PC12 cells	Neuroprotective	[41]
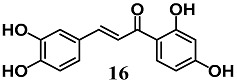 (*E*)-1-(2,4-dihydroxyphenyl)-3-(3,4-dihydroxyphenyl)prop-2-en-1-one (Butein)	30 μM	Obesity and Type 2 diabetes	Adipocyte 3T3-L1 cells	Antioxidant	[42]

^a^ Topical administration; ^b^ oral administration; ^c^ intraperitoneal administration; ^d^ the authors evaluated a surrogate marker of Keap1-Nrf2-ARE pathway activation.

**Table 2:** Synthetic chalcones as Nrf2 inducers.

Compound	Active Concentration Against Nrf2	Target Disease	Study Model	Activity	Ref.
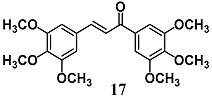 (*E*)-1,3-bis(3,4,5-trimethoxyphenyl)prop-2-en-1-one	30 μM	Inflammatory disease	Macrophage RAW 264.7 cells	Anti-inflammatory	[43]
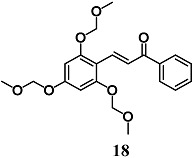 (*E*)-1-phenyl-3-(2,4,6-tris(methoxymethoxy)phenyl)prop-2-en-1-one	20 μM	Inflammatory intestinal disease	Intestinal HT-29 cells	Anti-inflammatory	[44]
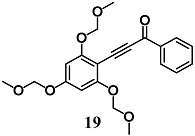 1-phenyl-3-(2,4,6-tris(methoxymethoxy)phenyl)prop-2-yn-1-one	2 μM	Inflammatory disease	Macrophage RAW 264.7 cells	Anti-inflammatory	[45]
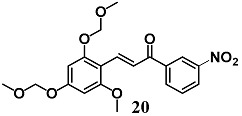 (*E*)-3-(2-methoxy-4,6-bis(methoxymethoxy)phenyl)-1-(3-nitrophenyl)prop-2-en-1-one	2 μM	Inflammatory disease	Macrophage RAW 264.7 cells	Anti-inflammatory	[46]
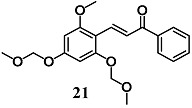 (*E*)-3-(2-methoxy-4,6-bis(methoxymethoxy)phenyl)-1-phenylprop-2-en-1-one (MBMC)	2 μM	Inflammatory disease	Macrophage RAW 264.7 cells	Anti-inflammatory	[47]
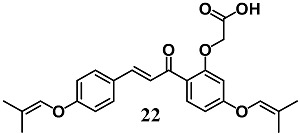 (*E*)-2-(5-((2-methylprop-1-en-1-yl)oxy)-2-(3-(4-((2-methylprop-1-en-1-yl)oxy)phenyl)acryloyl)phenoxy)-acetic acid (Sofalcone)	20–50 μM	Gastric ulcer	Gastric epithelial RGM-1 cells	Antiulcer	[48]
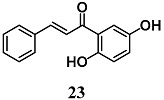 (*E*)-1-(2,5-dihydroxyphenyl)-3-phenylprop-2-en-1-one	10–20 μM	Oxidative stress	MCF-7/AREc32	Antioxidant	[49]
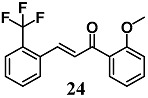 (*E*)-1-(2-methoxyphenyl)-3-(2-(trifluoromethyl)phenyl)prop-2-en-1-one	10–20 μM ^a^ 50 mg/Kg ^a,b^	Inflammatory disease	Bronchial epithelial Beas-2B cells Mice	Anti-inflammatory	[50]
	400 mg/Kg ^b^	Allergic asthma	Mice	Antioxidant and anti-inflammatory	[51]
	5 μM 400 mg/Kg ^b^	Systemic sclerosis	Fibroblasts Mice	Antioxidant	[52]
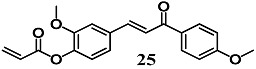 (*E*)-2-methoxy-4-(3-(4-methoxyphenyl)-3-oxoprop-1-en-1-yl)phenyl acrylate	10 μM ^a^	Oxidative stress	Neuronal PC12 cells	Antioxidant	[53]
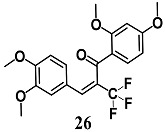 (*E*)-1-(2,4-dimethoxyphenyl)-3-(3,4-dimethoxyphenyl)-2-(trifluoromethyl)prop-2-en-1-one 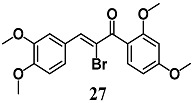 (*Z*)-2-bromo-1-(2,4-dimethoxyphenyl)-3-(3,4-dimethoxyphenyl)prop-2-en-1-one 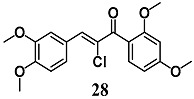 (*Z*)-2-chloro-1-(2,4-dimethoxyphenyl)-3-(3,4-dimethoxyphenyl)prop-2-en-1-one	3.13 μM	Inflammatory disease	MCF-7/AREc32 cells	Anti-inflammatory	[54,55]
	6.25 μM				
	6.25 μM				
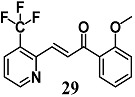 (*E*)-1-(2-methoxyphenyl)-3-(3-(trifluoromethyl)pyridin-2-yl)prop-2-en-1-one	5 μM ^a^ 400 mg/Kg ^a,b^	Oxidative stress	Bronchial epithelial Beas-2B cells Mice	Antioxidant	[56]
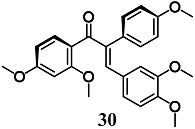 (*E*)-1-(2,4-dimethoxyphenyl)-3-(3,4-dimethoxyphenyl)-2-(4-methoxyphenyl)prop-2-en-1-one	30 μM	Inflammatory disease	Macrophage RAW264.7 cells	Antioxidant, cytoprotective and anti-inflammatory	[57,58]
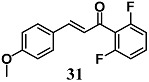 (*E*)-1-(2,6-difluorophenyl)-3-(4-methoxyphenyl)prop-2-en-1-one	10 μM 20 mg/kg ^b^	Diabetic cardiomyopathy	Cardiomyocyte H9c2 cells Mice	Anti-inflammatory and cytoprotective	[59]
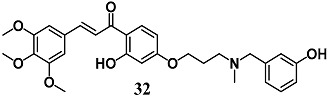 (*E*)-1-(2-hydroxy-4-(3-((3-hydroxybenzyl)(methyl)amino)propoxy)phenyl)-3-(3,4,5-trimethoxyphenyl)prop-2-en-1-one	10 μM ^a^	Alzheimer’s disease	Neuronal SH-SY5Y cells	Neuroprotective activity	[60]
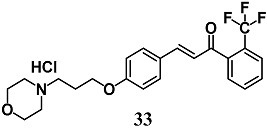 (*E*)-3-(4-(3-morpholinopropoxy)-phenyl)-1-(2-(trifluoromethyl)phenyl)-prop-2-en-1-one hydrochloride	3 μM 30 mg/kg ^a^	Parkinson’s disease	Dopaminergic CATH.a cells Mice	Neuroprotective	[61]
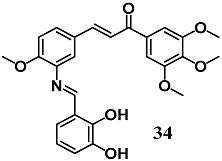 (*E*)-3-(3-(((*E*)-2,3-dihydroxybenzylidene)amino)-4-methoxyphenyl)-1-(3,4,5-trimethoxyphenyl)prop-2-en-1-one	5–10 μM 5–20 mg/kg ^b^	Diabetic cardiomyopathy	Cardiomyocyte H9c2 cells Mice	Antioxidant and anti-inflammatory	[62]

^a^ The authors evaluated a surrogate marker of Keap1-Nrf2-ARE pathway activation; ^b^ oral administration.
